# Around the Hospitals

**Published:** 1892-05-14

**Authors:** 


### Around the Hospitals.

**The Convalescent Home at Coatham**, Redcar, Yorkshire, which is for poor and deserving persons recovering from sickness, and requiring a change of air and sea-bathing, has now accommodation for 180 patients. The home was founded by the late Rev. John Postlethwaite, at a cost of over £4,500, borne wholly by himself. Since then the institution has found many other liberal friends, which has rendered possible the extension from 50 to 180 beds. Patients are admitted from any part of England, and receive all benefits free of charge. Each annual £2 2s. subscriber may send one adult patient or two children. The duration of the stay is fixed at one month. The nursing is done by ladies, who give their services free of charge. The patrons and visitors of the institution include the Archbishop of Canterbury and the Bishops of Durham, Oxford, and Ripon.

**The Samaritan Free Hospital for Women and Children** have to report a very prosperous year. The new buildings have been thoroughly tested and approved of as vastly superior to those previously in use. Through the munificent bequest of the late Mr. James Nasmyth the site and building, costing over £32,000, have been paid for. Mr. Nasmyth's bequest amounted to £18,000. The largest ward in the hospital has been named in memory of him. Mrs. Nasmyth has since presented 22 oil paintings, by her husband, to be distributed over the wards of the hospital, and a further sum of £4,000 will be paid at some later date to the hospital from the same source. The institution has received several other large bequests and donations. The hospital has treated 572 in-patients during the year, and it is to be noted that two-fifths of these have come from outside the metropolis. The fact is a surprising one, and so large a tax upon a metropolitan hospital should be proportionately met from the provinces by a financial recognition, which, we fear, is not the case.

It is with regret that we learn that the **Royal Sea Bathing Infirmary for Scrofula**, Margate, has not improved its financial position since last year. The income for many years has been exceeded by the expenditure, and the capital, which it has been necessary to draw upon, has thus diminished from nearly £11,000 to £1,500. The infirmary is one of our oldest institutions, having completed its century of existence in 1891. The demand on the accommodation of the hospital does not diminish. The late Sir Erasmus Wilson was a generous benefactor to the infirmary, and added, at his own expense, four wards, chapel, great sea-water bath, &c., in 1882, when the number of beds was raised to 220. Other benefactions have fallen to the lot of the institution since that period, but the number of annual subscribers is very considerably below what could be desired. The church collections during the year have not exceeded £20 at any one church, a very small sum, we venture to think, for so frequented a watering place as Margate.

**The Grosvenor Hospital for Women and Children** is able to congratulate itself on the successful results of critical operations performed during the year. Whilst this success has been quite unprecedented, financially the institution has suffered in common with most other charities, though not to a large extent, and it is to be hoped the deficiency will not increase, having regard to the many friends interested in the hospital. In the death of Mr. W. H. Smith the hospital sustained a great loss. The excellent practice of in-patients' payments is carried out, these varying from five shillings to ten shillings per week. There are also three private wards attached to the hospital, and patients in these are required to pay not less than £2 2s. the week. The out-patients' department is largely attended, 1,715 cases having been treated during the present year.

